# Where is an experienced medical educator when you need one?

**DOI:** 10.15694/mep.2020.000161.1

**Published:** 2020-08-10

**Authors:** Ian Wilson, Pathiyil Ravi Shankar

**Affiliations:** 1 International Medical University

**Keywords:** Covid19, pandemic, adaption, medical education

## Abstract

This article was migrated. The article was marked as recommended.

## Letter

Dear Editor

Change is often threatening because we must quickly adopt new modes and means of doing a task which are often contrary to our long-held beliefs and perceptions. Rapid changes to education are occurring during the current corona virus disease-19 (COVID-19) pandemic when, in most places, face-to-face teaching and assessment are prohibited and teaching-learning and assessment must occur online ( anon, 2020).

While lectures and tutorials are seen as practical to be conducted online, many academics are not able to conceive of teaching clinical skills or conducting high stakes examinations online. For clinical academics who have little experience with the medical education literature it is hard to know where to begin. This is where the expertise of those in medical education is needed.

The International Medical University (IMU), of Malaysia has a dedicated, fully equipped centre for medical education which played an important role in sustaining teaching-learning and assessment during the ongoing pandemic. In Malaysia, government edict closed all university campuses and put a halt to any form of face-to-face teaching ( anon, 2020). Quite quickly online teaching was permitted and encouraged, and this model persists at the time of writing. This resulted in several international, national and local education sessions for staff being held online. The common topics designed to quickly build capacity among faculty members included creating quality presentations, facilitating sessions, teaching clinical skills and undertaking high stakes assessment including objective structured clinical examinations (OSCEs) online.

IMU has invested substantially in online learning and created an e-learning department, a learning management system and the software needed to offer courses online. The university had shifted many sessions to a blended format with resource persons facilitating sessions online with support from local rapporteurs during the recently concluded Ottawa -2020 conference. The challenge for the university was in shifting clinical teaching-learning and assessment online. The university had a well-equipped simulation centre and standardised patients for clinical teaching-learning.

Clinical teachers started exploring various solutions and began carefully considering options presented by educators. Standardised patients were surveyed as to whether they would be prepared to work online with students. A large proportion were, and they were particularly helpful with teaching communications skills and engagement in online sessions. Much use was made of existing videos and material on history taking and clinical examination available online. Clinical examination skills were demonstrated, and students recorded themselves undertaking such skills for formative assessment and feedback by clinician teachers. Finding family members willing to be used as “patients” was not always possible. Over the last two months the clinical teachers developed a significant repertoire of techniques for teaching clinical skills. In preparing for this, teaching staff had to be prepared to work with students with poor internet connection ( Jalli, n.d.). This led to a focus on asynchronous presentations.

This process will enable students to return to the clinical setting with some ‘developed’ skills and will reduce the load of material that needs to be ‘assimilated’ in short intense teaching sessions once the situation improves. This is particularly important for those medical students graduating at the end of this year and who will do their internship next year.

Again, the process of running examinations online, particularly high stakes examinations was seen as difficult, if not impossible. While most were aware of proctoring systems for online examinations, the cost and student resistance ( Zhou, 2020) were unexpected challenges. Promotion of open book examinations with staff workshops exploring the processes involved went someway to overcoming the problem.

The combination of time-limited open-book examinations exploring higher-order cognitive skills, with a code of conduct and submission of examination material through a system such as Turn-it-in resolved many, but not all concerns. There is still a desire by some clinicians to defer all examinations until after the crisis when ‘traditional’ examinations with which they are familiar and in which they have confidence can be undertaken.

In working through the challenges brought by the COVID-19 pandemic we made use of the experience of educators in different contexts. Experience with, for example, remote rural supervision in Australian General Practice training ( Wearne, 2005), experience in the Malaysian and international contexts among others. We used the recording of patient-doctor consultations on tablets or smart phones for teaching students and trainee practitioners. Students submitted videos of consultations online for feedback and assessment from clinical preceptors. The preceptors with help from the educators have also developed a model for undertaking online OSCEs.

Addressing the crisis for the benefit of the students requires drawing on the experiences of staff and colleagues, adapting them for use in the current situation and finally disseminating them to staff who will use them. Studying, and adapting best practices from the literature can be helpful. The presence of an adequately staffed and equipped centre for education, resources for online learning and an open system of communication between faculty members, educators, educational technologists, administrators, and support staff involved is vitally important ( Varpio *et al.*, 2017).

## Notes On Contributors

Ian Wilson is an experienced medical educator in both undergradute and postgraduate fields. He has recently started working for the International Medical University as director of IMU Centre for Education. ORCID: https://orcid.org/0000-0003-2603-2660

Ravi Shankar is an experienced medical educator, predominantly in undergraduate medciine. He has particular expertise in teaching pharmacology.

## Declarations

The author has declared that there are no conflicts of interest.

## Ethics Statement

Ethical approval was not required for this letter because it is not reporting research findings.

## External Funding

This article has not had any External Funding
