# Supplementary material for: Where is an experienced medical educator when you need one?
Source: MedEdPublish (2016). 2020 Aug 10;9:161. [Version 1] doi: 10.15694/mep.2020.000161.1 (PMC10702643; doi:10.15694/mep.2020.000161.1)
Supplement: Peer review history

# Reviewer report: Reviewer response for version 1

**DOI:** 10.21956/mep.20123.r31045

**Published:** 2020-08-27

**Authors:** Wai Ching Lee

**Affiliations:** 1 Sengkang General Hospital

This review has been migrated. The reviewer awarded 4 stars out of 5 Interesting article, agree that in times of change - educators need to adapt fast and well to achieve the necessary learning objectives to benefit the students. It is very heartening to see that your university is immensely supportive to provide, train and grow the area of e-learning where in some areas it would be quite a big challenge.

Reviewer Expertise:

NA

No decision status is available

# Reviewer report: Reviewer response for version 1

**DOI:** 10.21956/mep.20123.r31048

**Published:** 2020-08-25

**Authors:** Neslihan Onder-Ozdemir

**Affiliations:** 1 The University of Sheffield

This review has been migrated. The reviewer awarded 4 stars out of 5 Thank you sharing your experience and perceptions regarding medical education during COVID-19. Indeed, with the novel coronavirus (COVID-19), the education system and the delivery of courses have changed worldwide. We should adapt to these changes, which will require some time, patience and collaboration. The letter above, written by Ian Wilson and Pathiyil Ravi Shankar, provides us with a picture in a medical university like a reflection report that shares first-hand experience and perceptions during COVID-19. Thus, these reflections may motive the medical teachers while delivering education during COVID-19. note: Some typos that can be corrected: Under the title Notes On Contributors:1- the word "undergradute" should be written as "undergraduate" (adding the letter "a" before the letter "t".)2- the word "medciine" should be written as "medicine" (through removing the second "i")

Reviewer Expertise:

NA

No decision status is available

# Reviewer report: Reviewer response for version 1

**DOI:** 10.21956/mep.20123.r31049

**Published:** 2020-08-24

**Authors:** Tharin Phenwan

**Affiliations:** 1 University of Dundee

This review has been migrated. The reviewer awarded 4 stars out of 5 I enjoy reading this letter and can relate to these challenges that we are facing. There are a lot that we need to adapt and improve, especially with asynchronous delivery methods i.e. how do we ensure that students can access the materials and engage as we want since some students do not have sufficient learning equipment. The lack of access to "patients" is another concern that we need to address and I am looking forward to the update from you.

Reviewer Expertise:

NA

No decision status is available

# Reviewer report: Reviewer response for version 1

**DOI:** 10.21956/mep.20123.r31047

**Published:** 2020-08-15

**Authors:** Sateesh Babu Arja

**Affiliations:** 1 Avalon University School of Medicine

This review has been migrated. The reviewer awarded 4 stars out of 5 I really enjoyed reading this letter to the editor. This article is timely under the current circumstances. This paper reflects what we are doing. I teach clinical skills for the first-year and second-year medical students. We are using standardized patients teaching clinical skills online. We are also encouraging students to practice history taking skills and physical examination on their family members and friends. As authors mentioned relatives and friends are not available for all students. We are also involved in conducting online OSCE examinations for final year medical students which is a graduation requirement. The outcomes are not known yet as authors mentioned. But as a medical educator for fifteen years, I feel that we are doing the best under the current circumstances. But as we go on we learn from our experiences. Thank you.

Reviewer Expertise:

NA

No decision status is available

# Reviewer report: Reviewer response for version 1

**DOI:** 10.21956/mep.20123.r31044

**Published:** 2020-08-12

**Authors:** Balakrishnan Nair

**Affiliations:** 1 Centre for Medical Professional Development and University of Newcastle

This review has been migrated. The reviewer awarded 4 stars out of 5 In these testing times, when students can not learn from patients , we have to be innovative. Medical educators all over the world are struggling to teach and assess senior medical students . We need them to be workforce -ready for next year . So this article is timely and provides some helpful advice . Evaluation from students ,and standardised patients would have been valuable . Hopefully this will be the next paper from this team.

Reviewer Expertise:

NA

No decision status is available

# Reviewer report: Reviewer response for version 1

**DOI:** 10.21956/mep.20123.r31046

**Published:** 2020-08-11

**Authors:** Shazia Iqbal

**Affiliations:** 1 Alfarabi College of Medicine Riyadh, KSA

This review has been migrated. The reviewer awarded 4 stars out of 5 The title of this letter just grabbed my attention because its the right time to engage medical educators for the sustainability of pedagogical changes in the current scenario. The authors have very well highlighted the current challenges and ongoing rapid adaptations in medical education management during the COVID-19 crisis. International Medical University (IMU) has well managed the situation through a fully equipped center for medical education and e-learning department. However, the adjustments in distance clinical teaching and online assessments are extremely difficult, especially during the final year of the medical program. Moreover, the applicability is a great challenge in low resource countries in terms of massive investment in the development of e-learning units. We still need some practical and concrete solutions especially challenges related to clinical training and OSCE.Authors' perspectives about the engagement of medical educators will not only be valuable to share distance teaching and online assessments but also be beneficial for improving the quality of medical education at the undergraduate level. The idea of the recording of patient-doctor consultations on tablets or smartphones for teaching students and trainee practitioners is very practical. I would suggest clinical academic faculty and medical educators to apply and test these adaptive measures mentioned in this letter. Good Luck

Reviewer Expertise:

NA

No decision status is available
